# Supplementary material for: Leveraging AI to Explore Structural Contexts of Post-Translational Modifications in Drug Binding
Source: bioRxiv. 2025 Mar 20:2025.01.14.633078. Preprint. [Version 2] doi: 10.1101/2025.01.14.633078 (PMC11956905; doi:10.1101/2025.01.14.633078)
Supplement: Supplement 4 [file media-4.pdf]

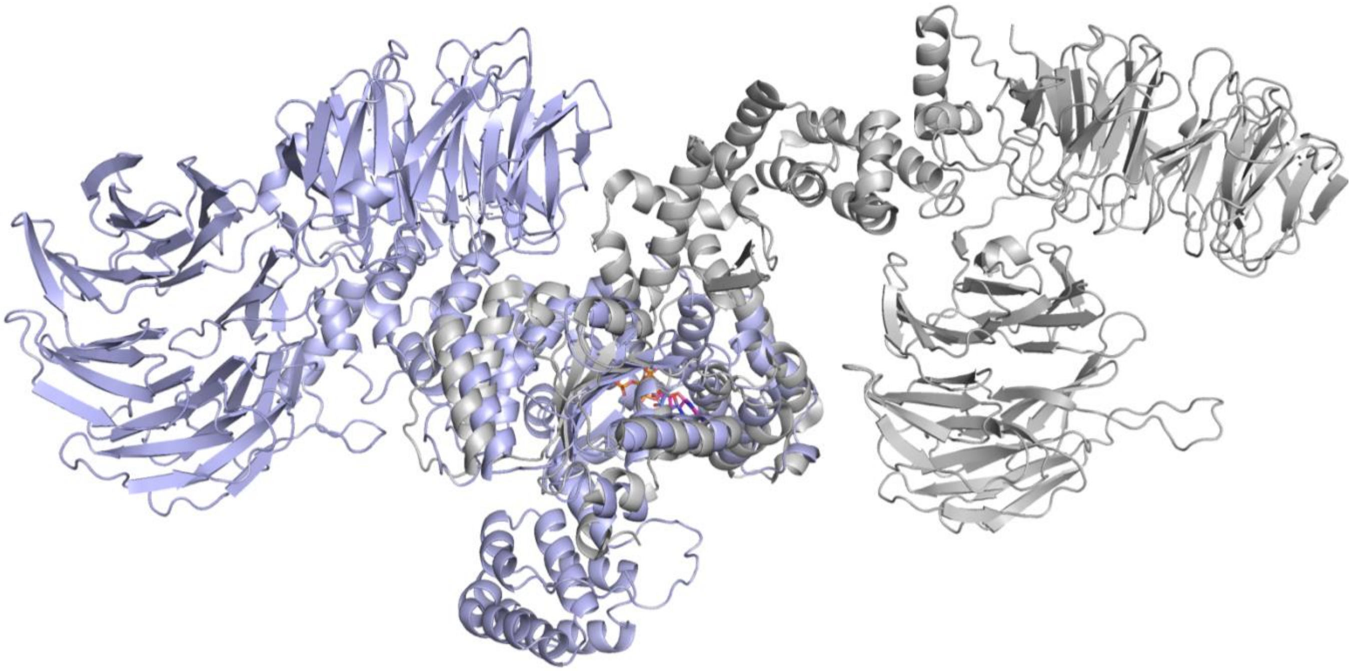

Supplementary Figure 1. Example of mismatch of domains orientation between model and experimental structure. Structure of apoptotic protease-activating factor 1 (PDB: 3J2T, shown in grey). AlphaFold3 model shown in light blue.

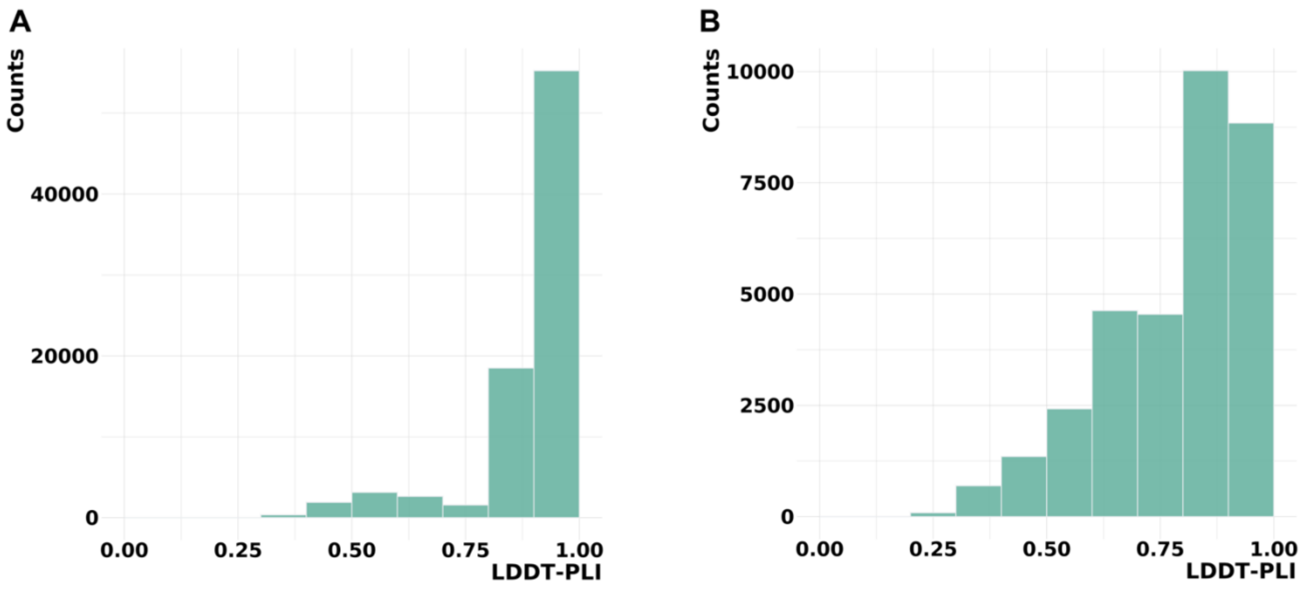

Supplementary Figure 2. LDDT-PLI scores distribution. (A) AlphaFold3 models vs experimental PDB structures. **(B)** RoseTTAFold All-Atom models vs experimental PDB structures.

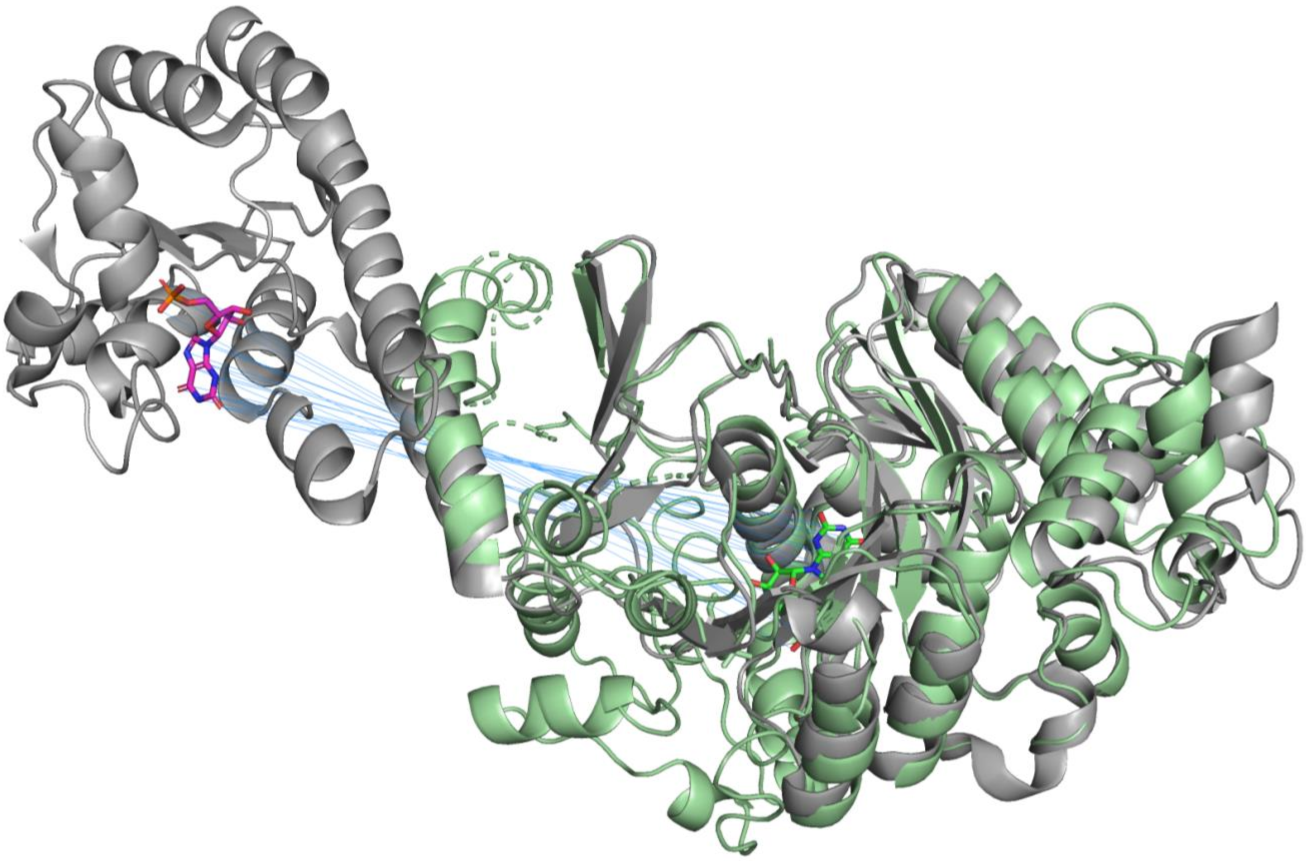

Supplementary Figure 3. Structure of Aminoimidazole-4-carboxamide ribonucleotide transformylase (PDB: 1PL0, shown in grey). RFAA model shown in light green. Ligand from experimental structure shown in magenta. Alignment lines between experimental and modelled ligands shown in blue.

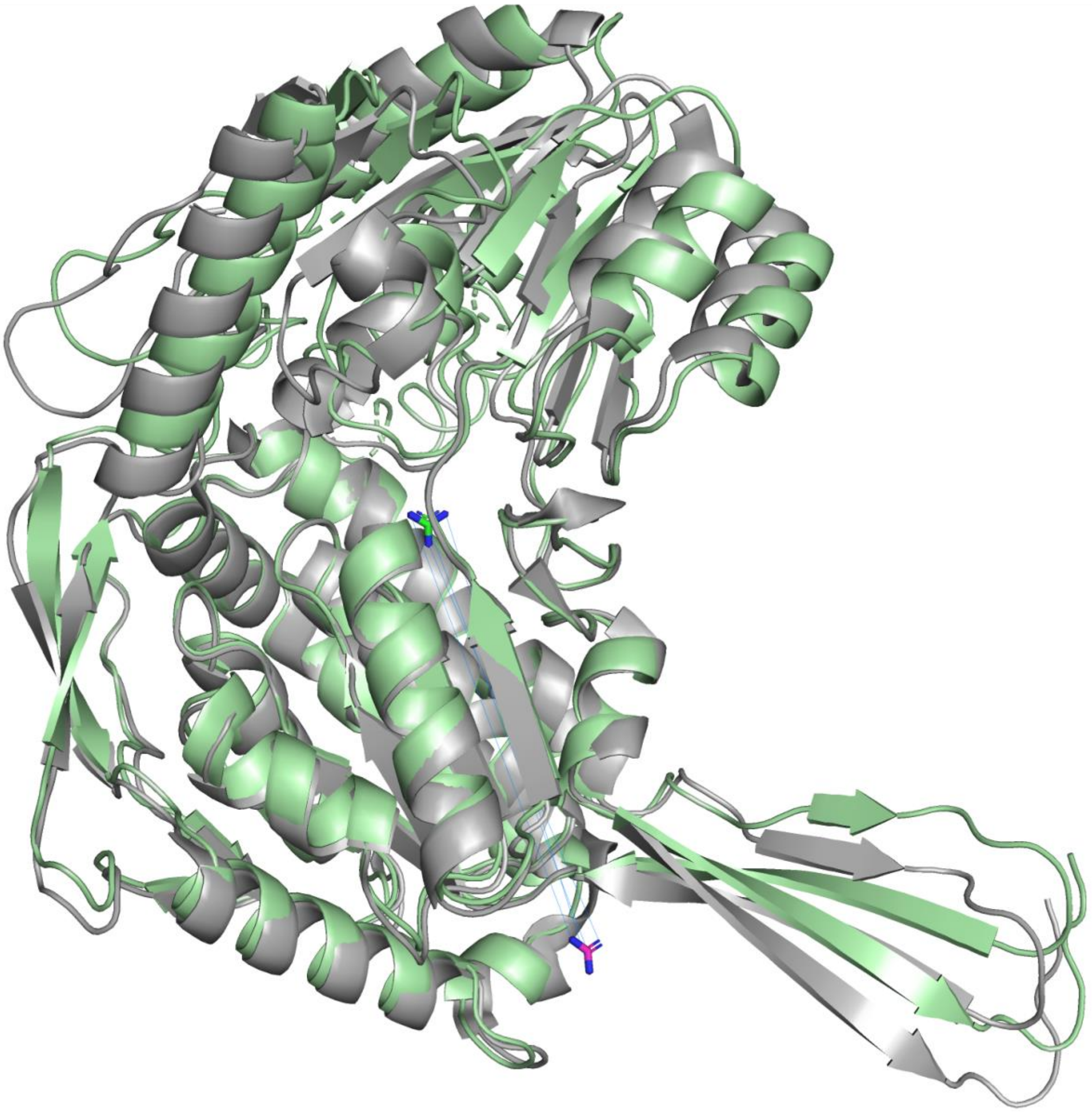

Supplementary Figure 4. Structure of mitochondrial aldehyde dehydrogenase (PDB: 3N80, shown in grey). RFAA model shown in light green. Ligand from experimental structure shown in magenta. Alignment lines between experimental and modelled ligands shown in blue.
